# Supplementary material for: Hanensula anomala isolated from the Berkeley Pit, Butte, MT, is a metal-specific extremophile
Source: Microbiol Spectr. 2024 Aug 20;12(10):e00444-24. doi: 10.1128/spectrum.00444-24 (PMC11448421; doi:10.1128/spectrum.00444-24)
Supplement: Figure S1 legend — PCR amplification from genomic H. anomala DNA. [file spectrum.00444-24-s0001.docx]

**Supplementary Figure 1. PCR amplification from genomic *H. anomala* DNA.** Amplified bands from genomic DNA. Lane 2: ITS3, ITS4; Lane 3: NS-1, NS-8; Lane 4: NS-1, NS-8A; Lane 5: NL-1, NL-4; Lane 6: NL-3A, NL-7AR; Lane 10: NL-E27, NL-11R; Lane 11: NL-G19A, NL-13R.
