# Supplementary figures and images for: Hanensula anomala isolated from the Berkeley Pit, Butte, MT, is a metal-specific extremophile
Source: Microbiol Spectr. 2024 Aug 20;12(10):e00444-24. doi: 10.1128/spectrum.00444-24 (PMC11448421; doi:10.1128/spectrum.00444-24)

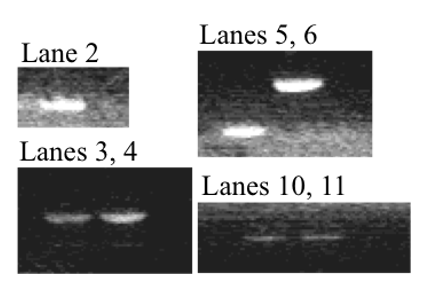

Supplement: Supplemental figure — Fig. S1. [file spectrum.00444-24-s0002.tiff]

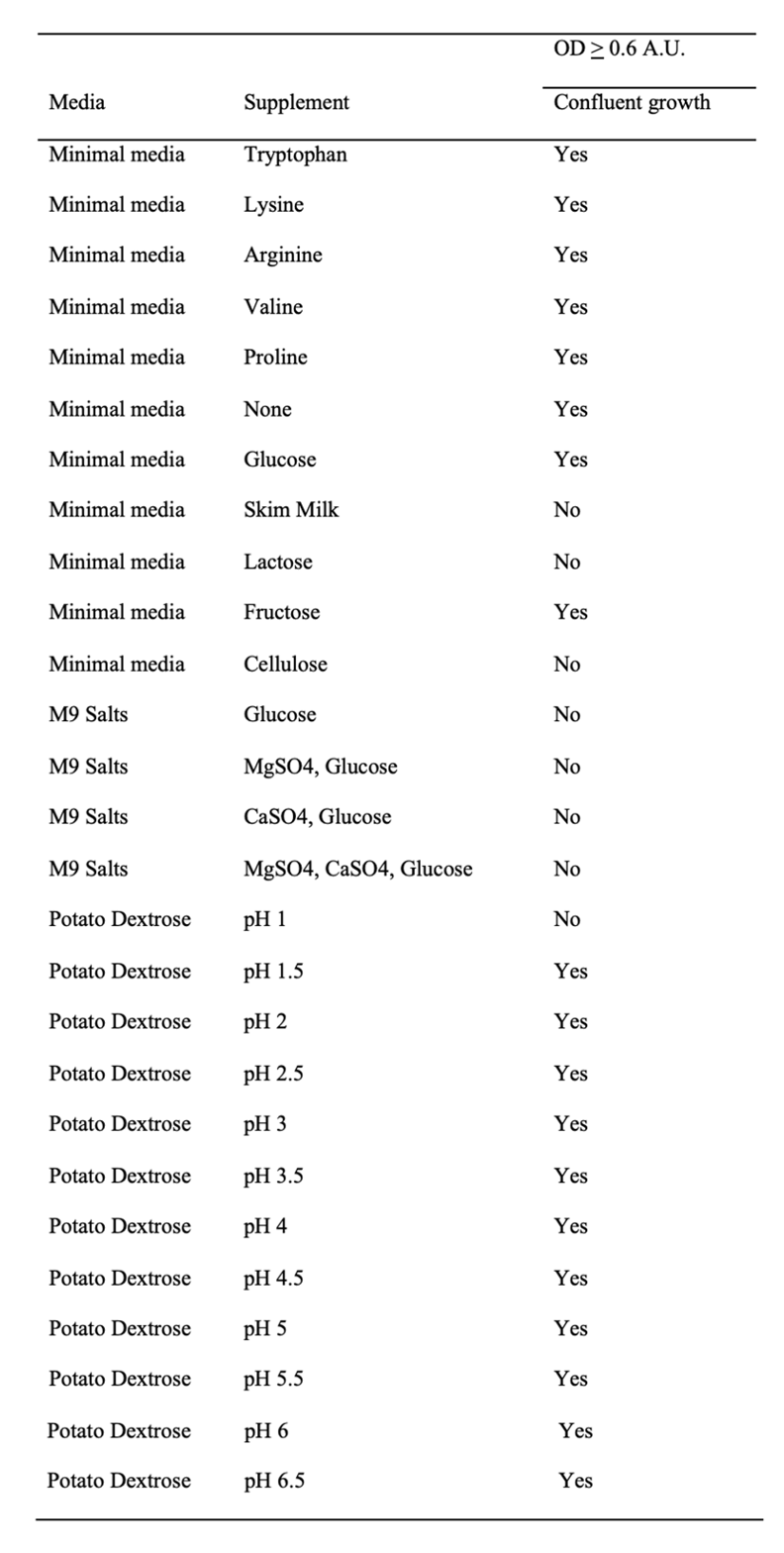

Supplement: Supplemental table — Table S1. [file spectrum.00444-24-s0004.tiff]
