# Supplementary material for: Hanensula anomala isolated from the Berkeley Pit, Butte, MT, is a metal-specific extremophile
Source: Microbiol Spectr. 2024 Aug 20;12(10):e00444-24. doi: 10.1128/spectrum.00444-24 (PMC11448421; doi:10.1128/spectrum.00444-24)
Supplement: Table S1 legend — Growth tests. [file spectrum.00444-24-s0003.docx]

**Supplementary Table 1.** Growth tests observed at 0.6 A.U. cutoff, supplements for each sample

are either metal, solute, amino acid or variable pH with sulfuric acid
